# A study of soft tissue sarcomas after childhood cancer in Britain

**DOI:** 10.1038/sj.bjc.6603908

**Published:** 2007-07-24

**Authors:** H C Jenkinson, D L Winter, H B Marsden, M A Stovall, M C G Stevens, C A Stiller, M M Hawkins

**Affiliations:** 1 Department of Paediatric Oncology, Birmingham Children's Hospital, Steelhouse Lane, Birmingham B4 6NH, UK; 2 Department of Public Health and Epidemiology, University of Birmingham, Birmingham B15 2TT, UK; 3 Department of Paediatric Pathology, Royal Manchester Children's Hospital, Manchester M27 4HA, UK; 4 Department of Radiation Physics, The University of Texas, MD Anderson Cancer Center, Houston, TX, USA; 5 Institute of Child Life and Health, University of Bristol, Bristol BS2 8AE, UK; 6 Childhood Cancer Research Group, Department of Paediatrics, University of Oxford, Oxford OX2 6HJ, UK

**Keywords:** second soft tissue sarcoma, childhood cancer

## Abstract

Among 16 541 3-year survivors of childhood cancer in Britain, 39 soft tissue sarcomas (STSs) occurred and 1.1 sarcomas were expected, yielding a standardised incidence ratio (SIR) of 16.1. When retinoblastomas were excluded from the cohort, the SIR for STSs was 15.9, and the cumulative risk of developing a soft tissue tumour after childhood cancer within 20 years of 3-year survival was 0.23%. In the case–control study, there was a significant excess of STSs in those patients exposed to both radiotherapy (RT) and chemotherapy, which was five times that observed among those not exposed (*P*=0.02). On the basis of individual radiation dosimetry, there was evidence of a strong dose–response effect with a significant increase in the risk of STS with increasing dose of RT (*P*<0.001). This effect remained significant in a multivariate model. The adjusted risk in patients exposed to RT doses of over 3000 cGy was over 50 times the risk in the unexposed. There was evidence of a dose–response effect with exposure to alkylating agents, the risk increasing substantially with increasing cumulative dose (*P*=0.05). This effect remained after adjusting for the effect of radiation exposure.

Survival after childhood cancer has greatly improved over the last three decades and most recent figures indicate that about 75% of children diagnosed with cancer are likely to survive at least 5 years (Toms, 2004). Thus a growing number of survivors, estimated at 1 in every 1000 young adults (Hawkins and Stevens, 1996), are at risk of various adverse late effects of both the cancer and its treatment. The well-recognised increased risk of a second malignant neoplasm (SMN) (Meadows *et al*, 1985) may represent the greatest challenge to long-term survival (Robison and Mertens, 1993). Further study of their causes, which include both exposure to chemotherapy (CT), radiotherapy (RT) and genetic predisposition (Kony *et al*, 1997), requires follow-up of large numbers of survivors with a wide spectrum of treatments.

Soft tissue sarcoma (STS) represents an important risk of SMN following childhood cancer, particularly heritable retinoblastoma (Draper *et al*, 1986; Hawkins *et al*, 1987; Westermeier *et al*, 1998; Menu-Branthomme *et al*, 2004). The one published case–control study of STS following childhood cancer demonstrated an independent association with exposure to RT and CT with procarbazine (Menu-Branthomme *et al*, 2004).

We here examined the incidence of STS in a population-based cohort of 3-year survivors of childhood cancer in Britain, and explored their aetiological factors in a much larger case–control analysis than previously reported. For present purposes, STS occurring as an SMN will be referred to simply as STS.

## MATERIALS AND METHODS

### Cohort study

A cohort of 3-year survivors of childhood cancer was selected from the National Register of Childhood Tumours (NRCT), a population-based national register covering the whole of Great Britain, which is maintained by the Childhood Cancer Research Group (CCRG), at the University of Oxford. The register is notified of all cancers occurring in individuals below 15 years through the national cancer registration system in Britain, which was established in 1962. In addition, a complementary series of 3-year survivors of childhood cancer diagnosed before this date was constructed from case lists obtained from individual hospitals and cancer registries.

Soft tissue sarcomas were ascertained by several methods. First, cohort members were ‘flagged’ at the National Health Service Central Registers (NHSCR), a system that provides automatic notification of the registration of death or further cancer in these individuals (Hawkins and Swerdlow, 1982). At the time of finalising the cohort, virtually all cancer registrations for 1990 and earlier years had been fully processed at NHSCR and notified to researchers. Second, a series of postal questionnaires enquiring about any STS were sent to the family doctors of the survivors over the period 1982 to 1990, providing an independent source of ascertainment for 82% of the person-years of follow-up. Finally, the CCRG receives all death certificates, which mention neoplasia in patients aged less than 20 years in Britain. These are routinely checked through family doctor and hospital notes to identify multiple primary tumours. The cohort consisted of all malignant neoplasms diagnosed before 1988, aged less than 15 years and subsequently survived at least 3 years. Soft tissue sarcomas were included if they were diagnosed before the study end point of 31 December 1990, after which the completeness of ascertainment is uncertain.

### Pathological criteria

For each individual in the cohort, the first primary tumour was classified according to the International Classification of Diseases for Oncology (ICDO) (World Health Organization, 1976), and subsequently categorised into special diagnostic groups (Birch and Marsden, 1987). Soft tissue sarcomas were classified according to ICDO (World Health Organization, 1976). Soft tissue sarcomas were also classified by ICD-9 (World Health Organization, 1979) as general population incidence figures are available for this tumour classification.

### Statistical methods

Statistical tests and confidence intervals were based on the assumption that the observed numbers of cancers followed a Poisson distribution with mean equal to the expected number of cases. Person-years at risk were accumulated from entry into the study (at 3-year survivorship) until exit (defined as diagnosis of STS, death, emigration or study end point of 31 December 1990, whichever was first). The standardised incidence ratio (SIR) is the ratio of the observed to the expected number of cancers within the cohort. Expected numbers were estimated by accumulating person-years at risk within specific categories defined by 5-year age groups, sex and single calendar year, and then multiplying these by the general population incidence rate of malignant disease within the corresponding category (Breslow and Day, 1987; Office of National Statistics, 1999). The additive excess risk is the difference between the observed and the expected number of STSs divided by the person-years at risk and multiplied by 1000, and is a measure of the excess number of cancers per 1000 survivors per year (Breslow and Day, 1987).

### Case–control study

Fifty-three cases of STS were identified as described above, of which 39 were diagnosed before 31 December 1990 and nested within the cohort study. The remaining 14 cases included here were diagnosed during 1991–1996.

For each case, we attempted to select four matched controls from the NRCT on the following criteria:

1. Age at first cancer (within 1 year of the age at diagnosis of the case);
2. Sex;
3. Histological diagnosis of the first cancer, classification codes I–XI (Birch and Marsden, 1987). Retinoblastomas were also matched as to whether heritable or non-heritable, the former including either bilateral disease or a family history; the remainder were defined as non-heritable;
4. Period of first cancer diagnosis – within the same 5-year period: 1925–1929, 1930–1934, 1935–1939, etc;
5. The interval survived free of STS from first cancer diagnosis to exit for the control needed to be greater than or equal to the interval from first cancer to SSTS in the matched case.

The exit date for each case was the date of STS, and for each control the date at diagnosis of the first cancer plus the interval from first cancer to second cancer in the matched case.

All cases of STS in the case–control study underwent independent pathological review; representative sections from both primary and secondary cancers were obtained wherever possible and the diagnosis confirmed by a paediatric pathologist. For each control, wherever possible, the original pathology report of the first cancer was obtained from the patient's medical records.

Medical information from cancer registry data, hospital and occasionally GP records for the cases covered CT and RT for the first cancer and for any recurrences up until STS diagnosis, together with family and personal history of genetic conditions or cancer.

### Radiation dosimetry

For those cases and controls receiving RT, the original prescription sheets, planning diagrams and treatment details were obtained. These were anonymised, then electronically scanned onto CDs and sent to the Department of Radiation Physics, MD Anderson Cancer Center in Houston, for detailed radiation dosimetry. For sarcomas within areas treated for the first tumour, standard radiation therapy data were used to estimate dose. For sarcomas outside such areas, doses were based on radiation measurements in water phantoms, applied to a three-dimensional mathematically described anthropomorphic phantom that simulated patients of various ages. Contributions from collimator scatter, head leakage and scatter within the patient were included in the final calculations (Stovall *et al*, 2006). Dose categories (to cases and controls) to the relevant soft tissue sites were as follows: 0.1–49 cGy, 50–999 cGy, 1000–2999 cGy and 3000 cGy and above.

### Chemotherapy

Chemotherapy details for each individual course or cycle of treatment were coded, including dates of the start and end of each course along with the name of each drug, the dose and route of administration, and whether given alone or with other cytotoxic therapy. Cytotoxic agents were grouped by mode of action, the commonest being alkylating agents, vinca alkaloids, anti-tumour antibiotics, antimetabolites and epipodophyllotoxins. The cumulative dose of alkylating agents received was divided into four categories of increasing exposure: 0.0–5999 mg, 6000–11 999 mg, and ⩾12 000 mg.

Numbers of cases and controls were compared using standard conditional logistic regression methods (Breslow and Day, 1980) using EGRET epidemiological software (1999). All tests of significance are two-tailed unless otherwise stated.

## RESULTS

### Cohort study

The cohort comprised 16 541 3-year survivors of childhood cancer diagnosed 1926–1987, and followed-up for a mean of 10 years (median: 7 years 7 months), with a mean age at diagnosis of 6 years 8 months (median: 5 years 10 months). A total of 39 STSs met the ICDO definition, and 17 of these were classified by ICD-9 as 171 compared to 1.1 expected from population incidence rates, giving an SIR of 16.1 (95% CI 9.4, 25.8). When retinoblastomas were excluded, the SIR for STS was 15.9 (95% CI 8.9, 26.2). The first cancer diagnoses in cases of STS were Wilms' (4), brain and CNS (4), heritable retinoblastoma (2), Hodgkins' (2) and 1 each of neuroblastoma, acute lymphoblastic leukaemia, bone cancer, STS and renal cell carcinoma.

The highest risks of STS followed primary Wilms' tumour and heritable retinoblastoma with SIRs of 45.9 (95% CI 12.5, 117.4) and 41.3 (95% CI 5.0, 149), respectively. There was no evidence of significant heterogeneity or trend in the SIRs when analysed by duration of follow-up from the first cancer, age at diagnosis of the first cancer and period of diagnosis. Similar analyses revealed evidence of heterogeneity in SIRs by treatment for the primary cancer, of borderline statistical significance (Table 1). No STSs were observed in patients exposed to neither RT nor CT, and the greatest risk (SIR=34.5) was after exposure to both RT and CT.

The cumulative risk of STS after childhood cancer (excluding retinoblastoma) within 20 years of 3-year survival was 0.23% (s.e.=0.07%) by the ICD-8 and -9 and 0.44% (s.e.=0.1%) using the ICDO.

### Case–control study

In all, 53 cases of STS were included, of which 39 were nested within the cohort study (25 cases male and 28 female). The mean interval between diagnoses of first cancer and STS was 16 years and 4 months (mean age at diagnosis of STS, 23 years). All original diagnoses were histologically confirmed, 43 cases (81%) being subject to central pathological review, 1 (2%) underwent central pathological review as part of a clinical trial and the remaining 9 (17%) had insufficient tissue available for histological verification of the original diagnosis. Thirty cases had 4 controls, 15 had 3, 6 had 2 and 2 had 1, a total of 179 controls.

### Treatment

Eight controls with missing information regarding RT or CT were designated ‘no record’. Using those exposed to neither RT nor CT as the reference or unexposed category (i.e. relative risk (RR)=1), all treatment categories (RT, CT and both) showed excess risks, significantly so with exposure to both RT and CT (*P*=0.02), with STS risk almost five times higher than in the unexposed group, a broad indication of treatment-related risk.

Most individuals were treated with RT either alone or with CT (79% cases and 71% controls). There was an excess risk of STS in the exposed group (RR=1.6; 95% CI 0.7, 3.7) compared with the unexposed. Details of STS risks are shown in Table 2, including a significant dose– response relationship with increasing cumulative dose of radiation; the RR in the highest exposure category (3000 cGy and over) was 38 times that seen in the reference group (*P*<0.001), with evidence of heterogeneity (*P*<0.001) and linear trend (*P*<0.001) in risks across the different exposure categories. When the RR was adjusted for the effect of exposure to alkylating agents, by fitting a factor with four exposure levels, there remained a significant dose–response relationship, the risk in the highest exposure category being over 50 times that in the unexposed group.

Chemotherapy, received by 43% of cases and 34% of controls, was associated with a significantly elevated risk of STS (RR=3.1; 95% CI 1.1, 8.8). The results for alkylating agents are presented in Table 3. Univariate analysis showed a dose–response relationship (*P*=0.05) with risk highest in those exposed to over 12 000 mg alkylating agent (RR=4.73; *P*=0.043). The relative risk across the dose categories demonstrated evidence of a linear trend (*P*=0.05). After adjustment for radiation dose (five levels), there remained a significant linear trend across the dose categories (*P*=0.05). No dose–response relationship was found with vinca alkaloids, and other categories of drugs contained insufficient numbers for analysis.

The 14 STS cases diagnosed after the study end point may not be representative of all cases if ascertainment was incomplete, thereby introducing potential bias of the risk assessment with RT or CT. An analysis excluding STS cases diagnosed after 1990 found excess risks of STS following all anti-cancer treatments, with a significant excess for patients receiving both RT and CT (*P*=0.03). In the highest RT exposure group (3000 cGy and over), risk was 37-fold (95% CI 3.3, 418) than in the unexposed group. However, the numbers were too small for the CT model to converge.

## DISCUSSION

The cohort analysis gives an overall SIR of developing an STS of 16.1, similar to that after excluding retinoblastomas as first cancer (SIR=15.9). Soft tissue sarcoma accounted for 5.8% of all SMNs and represented the second highest risk (SIR=16.1) after secondary bone cancer (SIR=41.1) (Jenkinson *et al*, 2004). The greatest STS risk followed Wilms' tumour (SIR=45.9) and heritable retinoblastoma (SIR=41.3), based on four and two cases respectively. Despite the high risk of STS compared with that in the general population, this equates to a reassuringly low absolute risk of 0.44%.

Whereas a strong association between heritable retinoblastoma and STS is well established (Draper *et al*, 1986; Eng *et al*, 1993; Wong *et al*, 1997), that with Wilms' tumour is less well known. A large cohort of Wilms' tumour survivors (5278) treated during 1969–1991 developed a number of both solid tumours (Breslow *et al*, 1995) and haematological second malignancies (Shearer *et al*, 2001). Of the 34 secondary solid tumours, 22 (65%) occurred within previously irradiated tissue and 13 (60%) of these were bone sarcomas or STS; STS risk was associated with both RT and CT (doxorubicin) (Breslow *et al*, 1995).

The overall cumulative risk of STS (based on ICD-8 and -9) within 20 years of a childhood cancer was 0.23%, lower than that (0.44%) based on ICDO, which included an additional 22 cases. The discrepancy between the 17 STS cases diagnosed by ICD-9 (in the cohort analysis) and the 39 cases classified by ICDO (in the case–control study) highlights an issue in classifying childhood cancer by site-based classification codes, as generally used in international comparisons. Thus ICD-8 and -9 coding misses STS of liver, kidney, breast, lung and bladder. For such reasons, a classification for childhood cancer was introduced which was based upon both morphology and site (Birch and Marsden, 1987), and this scheme was used to identify the cases included in the case–control study.

This, the largest case–control study of STS among survivors of childhood cancer, finds a highly significant dose–response relationship with RT, the risk increasing with increasing cumulative dose to soft tissue (trend *P*<0.001). This relationship persisted after adjusting for the effect of exposure to alkylating agents in a multivariate conditional logistic regression model. The adjusted risk to those exposed to 3000 cGy or more was over 50-fold (95% CI 6.0, 441) the risk in tissue unexposed to RT. A case–control study of 25 STS found that an increased risk was related to the square of the RTdose to the site at which the STS developed (Menu-Branthomme *et al*, 2004).

As the risks of secondary sarcomas of bone and soft tissue have often been considered together, it seems reasonable to compare the case–control findings reported here with those from previous studies of bone sarcomas after childhood cancer. In a nested case–control study of 64 cases of bone cancer, a strong dose–response relationship was found with radiation exposure (Tucker *et al*, 1987), while a similar pattern of risk was reported from a case–control study of 59 cases among 3-year survivors of childhood cancer (Hawkins *et al*, 1996). The fall in relative risk at the highest doses of RT may have been due to chance and reflect the wide confidence intervals, but it may also result from the ‘cell-killing’ phenomenon reported by a number of other workers (Boice *et al*, 1987). In a smaller case–control study of second bone cancers that also demonstrated an increasing risk with increasing cumulative dose of both RT and CT (mainly alkylating agents), the risk of second bone cancers was found to be a linear function of the local RT dose, with an excess relative risk of 1.8 per gray (Le Vu *et al*, 1998). The similarities between these studies of bone cancer and the present study of STS support our conclusion that risk increases with increasing cumulative dose of radiation.

A dose–response relationship was also found with alkylating agent CT. After adjusting for RT in a multivariate model, the risk to patients receiving over 12 g m^−2^ of alkylating agents was five times (95% CI 1.1, 30.6) that in the unexposed. Procarbazine, an alkylating agent, was also an independent risk factor for STS (Menu-Branthomme *et al*, 2004). Conclusions from case–control studies investigating second bone sarcomas have been very similar including a dose–response relationship (Tucker *et al*, 1987; Hawkins *et al*, 1996). Some evidence has been reported of anthracyclines as a potential STS risk factor (Neglia *et al*, 2001). Chemotherapy has also been implicated in excess risks of SMN after Hodgkin's disease (Kaldor *et al*, 1987; Tucker *et al*, 1988; Hancock *et al*, 1993; Cellai *et al*, 2001). The investigation of individual cytotoxic agents will require much larger numbers of exposed individuals and probably international collaboration.

## Figures and Tables

**Table 1:** Observed and expected numbers of sarcomas and SIRs for developing second soft tissue sarcomas after childhood cancer (excluding retinoblastoma) by treatment for the primary cancer

**Treatment received for the first cancer**	**Number entering risk interval**	**Observed**	**Expected**	**SIR**	**95% CI for SIR**
No RT or CT	2190	0	0.19		
CT only	1433	1	0.06	16.9	0.4, 94.1
RT only	3498	6	0.34	17.6	6.5, 38.3
Both RT and CT	5620	8	0.23	34.5	14.9, 68.0
Statistical test for heterogeneity in SIRs				*P*=0.08	

CI=confidence interval; CT=chemotherapy; RT=radiotherapy; SIRs=standardised incidence ratios.

**Table 2:** RRs of developing a second soft tissue sarcoma in relation to cumulative dose of radiation

	**Number of patients (median dose in cGy)**		**RR (95% CIs)**		**RR (95% CI)**	
**Dose of radiation (cGy) to site of second STS**	**Cases**	**Controls**	**Unadjusted**	***P*-value**	**Adjusted for alkylating agent exposure**	***P*-value**
No record	2	10				
0	11	55	1.0		1.0	
1–49	9 (12)	46 (11)	1.02 (0.36, 2.88)	0.97	1.28 (0.35, 4.63)	0.711
50–999	8 (180)	42 (180)	2.4 (0.59, 9.58)	0.22	3.73 (0.78, 17.7)	0.098
1000–2999	13 (1810)	16 (1605)	21.8 (3.3, 143.8)	0.0014	37.08 (4.45, 309.3)	<0.001
⩾3000	10 (3765)	10 (4000)	38.5 (5.19, 285.2)	<0.001	51.35 (5.97, 441.5)	<0.001
Likelihood ratio test for evidence of heterogeneity in RR over the five different exposure categories			*P*<0.001		*P*<0.001	
Likelihood ratio test for evidence of linear trend across the five different exposure categories			*P*<0.001		*P*<0.001	

CI=confidence intervals; RRs=relative risks; STS=soft tissue sarcoma.

Adjusted RRs and *P*-values were derived by simultaneously fitting a factor for alkylating agent exposure with four levels of exposure.

**Table 3:** RR of developing a second soft tissue sarcoma in relation to cumulative exposure to alkylating agents

	**Number of patients (median dose in mg)**		**RR (95% CI)**		**RR (95% CI)**	
**Cumulative dose of alkylating agents in mg m^−2^**	**Cases**	**Controls**	**Unadjusted**	***P*-value**	**Adjusted for radiotherapy exposure**	***P*-value**
No record	6	16				
0	36	128	1.0		1.0	
0–5999	2 (2413)	11 (3795)	0.85 (0.14, 5.12)	0.856	0.39 (0.04, 3.54)	0.403
6000–11 999	4 (9452)	15 (7839)	1.72 (0.29, 10.49)	0.554	2.33 (0.2, 27.32)	0.499
12 000 and above	5 (15 895)	9 (13 988)	4.73 (1.05, 21.36)	0.043	5.72 (1.07, 30.64)	0.042
Likelihood ratio test for evidence of heterogeneity in RR over the four different exposure categories			*P*=0.215		*P*=0.126	
Likelihood ratio test for evidence of linear trend across the four different exposure categories			*P*=0.05		*P*=0.05	

CI=confidence intervals; RR=relative risk.

Adjusted RRs and *P*-values were derived by simultaneously fitting a factor for radiation exposure with five levels of exposure.
